# Editorial: Electrochemistry of rechargeable aqueous metal-ion batteries: recent advances and future opportunities

**DOI:** 10.3389/fchem.2026.1789044

**Published:** 2026-01-26

**Authors:** Le Li, Runming Tao

**Affiliations:** 1 Shaanxi Key Laboratory of Industrial Automation, School of Mechanical Engineering, Shaanxi University of Technology, Hanzhong, China; 2 Independent Researcher, Darien, IL, United States

**Keywords:** aqueous metal-ion batteries, electrochemistry, energy storage mechanism, materials chemistry, rechargeable batteries

The core advantages of rechargeable aqueous metal-ion batteries lie in their high safety and low cost. Their application prospects primarily focus on two key directions: first, serving as a secure grid-scale energy storage solution for renewable energy integration and peak shaving; second, providing irreplaceable safety assurance in specialized fields, such as, wearable devices, medical implant electronics and emergency power supplies. Systems based on abundant elements like zinc and sodium also offer highly cost-competitive potential alternatives for consumer electronics and low-speed electric vehicles. Rechargeable aqueous metal-ion batteries have garnered significant attention primarily due to their inherently high safety from non-flammable aqueous electrolytes and the use of abundant, low-cost metals like zinc and iron. Simultaneously, they are considered good candidates for large-scale energy storage in the “post-lithium era”. Current research focuses on overcoming common challenges, such as, anode dendrites and hydrogen corrosion to advance their practical implementation. We are deeply honored to have compiled this Research Topic titled “*Electrochemistry of Rechargeable Aqueous Metal-ion Batteries: Recent Advances and Future Opportunities*” which vividly showcases all contemporary aspects of this research field, both from fundamental perspectives and applied viewpoints. This Research Topic features four invited articles covering the latest progress in rechargeable aqueous metal-ion battery research, encompassing all facets of the current landscape.

The first paper from Zhuang et al. employs density functional theory computation and Bader charge analysis to reveal the storage mechanism of aluminum (Al^3+^) at atomic scale. Its core finding is that Al^3+^ cations preferentially occupy the cobalt lattice sites rather than sulfur sites, representing a pseudo-epitactic substitution mechanism. The formation energy of this mechanism is 52.5% lower than that of substituting sulfur sites, providing an energetic rationale for the feasibility of Al^3+^ storage via a significant theoretical breakthrough. This study not only synthesized highly crystalline Co_9_S_8_ nanoparticles but also through structural characterization, explicitly identified agglomerates formed during synthesis as the key cause of severe electrode polarization and restricted ion diffusion kinetics. This work directly links the material morphological characteristics to the electrochemical performance, providing a clear direction for subsequent material optimization.

The second paper from Wu et al. creatively combined high-thermal-stability but poorly wettable polyimide (PI) with MXene, which exhibits excellent electrolyte affinity and ionic conductivity. Through a composite strategy, they simultaneously met the dual requirements of thermal safety and ion transport efficiency for separators. The composite separator not only retained PI’s inherent high-temperature resistance (operating normally at 120 °C) and flame-retardant properties but also significantly improved electrolyte wettability (contact angle of only 29°), reduced interfacial resistance (174 Ω) and enhanced mechanical strength (19.6 MPa) through the incorporation of MXene. These above resulted in substantially improved ion transport kinetics while ensuring safety. The batteries assembled with the proposed separator exhibit outstanding comprehensive performance: a 91% capacity retention over 200 cycles at 1 C (1C = ? mA/g), coupled with excellent rate capability. This demonstrates that the composite structure offers a promising and reliable solution to the challenge of balancing safety and cycle life in high-performance lithium-ion batteries.

The third article is a review paper from Ma et al., summarizing advanced nickel-based cathode materials for alkaline zinc-nickel batteries (AZNBs). This review precisely targets fundamental bottlenecks constraining the development of high-performance AZNBs, such as, sluggish redox chemistry and poor structural integrity. Innovatively grounded in fundamental principles of materials science, the review synthesizes diverse design strategies-including nanostructure optimization, defect engineering and ion doping-into coherent framework for enhancing redox kinetics and structural stability, thereby providing theoretical guidance for material design.

The last article written by Rao et al. describes the synthesis of NaCoPO_4_ using an ionothermal synthesis method based on ionic liquids. Compared to conventional high-temperature solid-state or hydrothermal methods, the proposed strategy typically offers milder conditions, lower costs and environmental friendliness, providing a novel and sustainable synthetic pathway for material preparation. Through this method, orthorhombic, uniformly distributed nanoscale particles (approximately 50 nm) were successfully synthesized. Such nanostructure facilitates shorter sodium ion diffusion paths and enlarges the electrode/electrolyte contact area, resulting to enhanced reaction kinetics. The synthesized NaCoPO_4_ was applied to aqueous rechargeable sodium-ion batteries At 0.1 C a good discharge specific capacity of 85 mAh g^-1^ with a 88% capacity retention rate over 100 cycles was achieved. This outcome provides a new effective option for developing safe and low-cost cathode materials for aqueous sodium batteries.

This research Research Topic features original studies encompassing all contemporary research aspects in the field of rechargeable aqueous metal-ion batteries. We express our profound gratitude to all authors for their outstanding contributions and for sharing the latest research findings in this Research Topic. Special thanks go to Journal Specialist Chen Wang for his dedicated assistance, valuable advice and secretarial support throughout the editorial process. We sincerely thank all reviewers for their timely and rigorous scrutiny of the manuscripts despite their busy schedules. Ultimately, if the content of this research Research Topic proves both practical and inspiring not only to experts but also to young researchers venturing into this exciting field, we will consider our endeavor a success.

